# Influence of salivary conditioning and sucrose concentration on biofilm-mediated enamel demineralization

**DOI:** 10.1590/1678-7757-2019-0501

**Published:** 2020-03-27

**Authors:** Hadeel M AYOUB, Richard L GREGORY, Qing TANG, Frank LIPPERT

**Affiliations:** 1 King Saud University Dental Health Department College of Applied Medical Sciences Riyadh Saudi Arabia King Saud University, Dental Health Department, College of Applied Medical Sciences, Riyadh, Saudi Arabia.; 2 Indiana University School of Dentistry Department of Biomedical Sciences and Comprehensive Care IndianapolisIndiana USA Indiana University, School of Dentistry, Department of Biomedical Sciences and Comprehensive Care, Indianapolis, Indiana, USA.; 3 Indiana University School of Medicine Department of Biostatistics IndianapolisIndiana USA Indiana University, School of Medicine, Department of Biostatistics, Indianapolis, Indiana, USA.; 4 Indiana University School of Dentistry Department of Cariology, Operative Dentistry and Dental Public Health IndianapolisIndiana USA Indiana University, School of Dentistry, Department of Cariology, Operative Dentistry and Dental Public Health, Indianapolis, Indiana, USA.

**Keywords:** Dental caries, Biofilms, Salivary pellicle, Saliva

## Abstract

The acquired pellicle formation is the first step in dental biofilm formation. It distinguishes dental biofilms from other biofilm types.

**Objective:**

To explore the influence of salivary pellicle formation before biofilm formation on enamel demineralization.

**Methodology:**

Saliva collection was approved by Indiana University IRB. Three donors provided wax–stimulated saliva as the microcosm bacterial inoculum source. Acquired pellicle was formed on bovine enamel samples. Two groups (0.5% and 1% sucrose–supplemented growth media) with three subgroups (surface conditioning using filtered/pasteurized saliva; filtered saliva; and deionized water (DIW)) were included (n=9/subgroup). Biofilm was then allowed to grow for 48 h using Brain Heart Infusion media supplemented with 5 g/l yeast extract, 1 mM CaCl_2_.2H_2_O, 5% vitamin K and hemin (v/v), and sucrose. Enamel samples were analyzed for Vickers surface microhardness change (VHN_change_), and transverse microradiography measuring lesion depth (L) and mineral loss (∆Z). Data were analyzed using two-way ANOVA.

**Results:**

The two-way interaction of sucrose concentration × surface conditioning was not significant for VHN_change_ (p=0.872), ∆Z (p=0.662) or L (p=0.436). Surface conditioning affected VHN_change_ (p=0.0079), while sucrose concentration impacted ∆Z (p<0.0001) and L (p<0.0001). Surface conditioning with filtered/pasteurized saliva resulted in the lowest VHN_change_ values for both sucrose concentrations. The differences between filtered/pasteurized subgroups and the two other surface conditionings were significant (filtered saliva p=0.006; DIW p=0.0075). Growing the biofilm in 1% sucrose resulted in lesions with higher ∆Z and L values when compared with 0.5% sucrose. The differences in ∆Z and L between sucrose concentration subgroups was significant, regardless of surface conditioning (both p<0.0001).

**Conclusion:**

Within the study limitations, surface conditioning using human saliva does not influence biofilm–mediated enamel caries lesion formation as measured by transverse microradiography, while differences were observed using surface microhardness, indicating a complex interaction between pellicle proteins and biofilm–mediated demineralization of the enamel surface.

## Introduction

Dental caries is a multifactorial disease, in which acid–producing bacteria, dietary carbohydrates, time, and a susceptible host contribute to the disease initiation and progression.^1^ The process starts when oral bacteria, present in an equilibrium state, ferment carbohydrates; this equilibrium shifts to increased populations of acidogenic (acid–producing) and aciduric (acid–tolerant) bacteria.^1^ The consistent presence of acid in the environment disrupts the mineral equilibrium of the exposed dental structures (i.e. enamel and/or dentin), and, therefore, leads to carious lesions.^1,2^

Dental biofilm has been defined as “matrix–enclosed microbial communities in which cells adhere to each other and/or to surfaces or interfaces.”^3^ Over 700 bacterial species are present in the oral cavity.^4^ They are in all oral hard and soft tissue structures. These bacterial aggregations usually produce and become enclosed in extracellular polymeric substance (EPS). The formation of dental biofilm (or dental plaque) consists of several steps, which start with the formation of the acquired pellicle, followed by the initial adhesion of planktonic bacteria to the pellicle layer by binding sites, subsequent maturation of the bacterial biofilm, and, finally, the dispersion of biofilm with detachment of cells/clusters of cells.^5^

The formation of the acquired pellicle is the first step in dental biofilm formation, and it is a unique step distinguishing it from other biofilm types.^5^ It consists of several interactions between various salivary glycoproteins, and their interaction with the tooth surface. These biochemical interactions are based on Gibbs law of free enthalpy^5,6^; they lead to the attachment of salivary glycoproteins to a surface (i.e. the enamel). The resulting formed layer is a protein–rich layer with binding sites; these sites are ready for early colonizers to attach.^6^

Based on this unique process, some studies suggested a new intervention to prevent biofilm formation: this intervention is in the form of preventing pellicle formation.^7^ Many microbial studies have explored and studied dental biofilm from many aspects using different cariogenic models.^8-11^ However, they omitted the step of surface conditioning by the formation of acquired pellicle. This leads to less clinical relevance, especially for this area of study (the significance of including the pellicle) has not been researched previously.

Acquired enamel pellicle (AEP) has been explored previously for its composition and function.^12-16^ Studies have explored pellicles and found differences between AEP formed *in vitro, in vivo*, and *in situ*. These studies have reported ultrastructural variations, intrinsic and extrinsic maturation variations, as well as variation in the AEP morphology. Studies have found that *in vitro* AEP were superior to *in vivo*, which contain higher amounts of proteins. They are also superior in the overall amounts produced (due to the difficulty in collecting *in vivo* AEP).^12-16^

In *in vitro* studies, the salivary pellicle can typically form before exposure to bacteria–containing media, resulting in biofilm formation. Several methods have been used to form a salivary pellicle.^17-19^ In general, the dental surface is exposed to saliva (sterilized, free from bacteria) for a specific amount of time (ranges from minutes to several hours) before being exposed to oral bacteria for biofilm formation.^17-19^ The significance of surface conditioning before biofilm growth (to allow the formation of acquired enamel pellicle) in studying biofilm models was not evaluated previously and, therefore, needs to be explored. Hence, this study aims to explore the influence of salivary conditioning before biofilm formation on enamel demineralization.

The hypothesis was: 1) a significant difference between filtered/pasteurized saliva, filtered saliva, and deionized water (DIW; negative control) as conditioning agents on biofilm–mediated enamel demineralization; and 2) a significant difference between 0.5% and 1% sucrose–supplemented growth media on enamel demineralization.

## Methodology

### Specimen preparation

Extracted bovine incisors were sectioned to obtain 5×5 mm enamel specimens using a Buehler Isomet^TM^ low-speed saw (Buehler, Ltd., Lake Bluff, IL, USA). Approximately 54 teeth were used to obtain 54 specimens. During preparation, the teeth were stored in deionized water with thymol. Using a Struers Rotopol 31/RotoForce 4 polishing unit (Struers Inc., Cleveland, PA, USA), all specimens were ground and polished to ensure flat parallel dentin/enamel surfaces. For the finishing process, the dentin side was ground using 500–grit silicon carbide grinding paper. Then, the enamel side was serially ground using 1,200, 2,400 and 4,000 grit papers. After that, specimens were polished using a 1–µm diamond polishing suspension on a polishing cloth to obtain a 5×5 mm polished enamel surface. All specimens were examined for cracks, white spots, or any other flaws that could exclude the specimen from the study, using Nikon SMZ 1500 stereomicroscope at ×20 magnification.

### Baseline measurement and experimental groups

All specimens were subjected to enamel surface microhardness test (VHN_sound_) to ensure standardization. A Vickers diamond identifier (Tukon 2100; Wilson-Instron, Norwood, MA, USA) was used with a load of 200 g for 15 s. Three indentations, approximately 100 µm apart, were placed on each specimen and averaged; the inclusion range was VHN_sound_ between 300-380. Specimens were divided into two groups, based on the sucrose concentration to which the biofilm/enamel surface was subjected (0.5% and 1% sucrose concentrations). Each group was divided further into three subgroups (n=9/subgroup), according to the nature of the salivary conditioning to the enamel surface before biofilm formation. The three conditions tested were: filtered/pasteurized saliva; filtered saliva; and deionized water (DIW; negative control).

### Salivary bacterial model

#### Biofilm model

After completing specimen preparation, specimens were mounted on the inside of a lid of a 6–well plate (FisherBrand, Fisher Scientific), with three specimens *per* well, using acrylic cubes to create an active attachment model and following a previously described protocol.^1,20^ The model was disinfected using 70% ethanol prior to bacterial and/or pellicle inoculation.^21^

#### Saliva collection

Ethical approval was obtained from the Indiana University (IUPUI) institutional review board (IRB #1406440799) for saliva collection. Wax–stimulated saliva samples from three adult donors were collected and pooled (approx. 50 mL/donor). The inclusion criterion considered healthy participants (no systemic diseases) with normal salivary flow and absence of active caries or periodontal disease. To ensure standardization, participants refrained from oral hygiene measures overnight. Before bacterial inoculation or freezing, the pooled saliva was tested for the presence of *Streptococcus mutans* and *Lactobacilli* using selective agars (MSSB and Rogosa agars, respectively). The results confirmed the presence of both species. A total of 5 mL of the pooled saliva and growth media mix (1:10 ratio) were incubated anaerobically overnight, then mixed with 10% glycerol and frozen immediately at −80ºC. This microcosm bacterial mix was used as the source for bacterial inoculum. The remaining pooled saliva was pasteurized as described below.

#### Saliva pasteurization

The collected, pooled saliva was diluted in sterile saline at 1:10 dilution. The diluted solution was filtered using Whatman filter paper to remove large debris. This filtered saliva was used to create the salivary pellicle in subgroups exposed to filtered saliva. For pasteurization, an additional sterilization step, pasteurization, was performed with the remaining filtered saliva, using a previously published protocol.^22^ Briefly, after the diluted solution first filtration, it was centrifuged to remove mucin and bacteria (10 minutes, 4ºC, 27,000× g). The supernatant was retained and pasteurized at 60ºC for 30 minutes, then recentrifuged for 10 minutes. The prepared saliva was stored in aliquots of 50 mL and frozen at −80°C for further use.

## Surface conditioning

All specimens were immersed in their corresponding solutions: filtered/pasteurized saliva, filtered saliva, or DIW as negative control. Specimens were incubated in their respective solution at 5% CO_2_ and 37ºC for 5 minutes to allow surface conditioning.

## Biofilm growth

Immediately after surface conditioning, specimens were transferred to a new, sterile 6–well plate containing growth culture media that was inoculated with the overnight bacterial culture (without washing the samples between the two steps). Microcosm biofilm was grown under anaerobic conditions at 37°C for 48 h. The growth media used to grow the biofilm was Brain Heart Infusion (BHI) broth, supplemented with 5 g/L yeast extract, 5% vitamin K and hemin (v/v) and supplemented with either 0.5% sucrose or 1% sucrose. After 48 h, the biofilm was collected by placing each specimen in an Eppendorf tube (containing 1 mL sterile saline), sonicated at 30 W for 10 seconds, and vortexed immediately for 10 seconds to completely detach biofilm from the enamel surface.

## Post-treatment analyses

### Surface microhardness change (VHNchange)

Post-treatment surface microhardness was measured following the same protocol used for the VHN_sound_. Three indentations were made at approximately 100 µm next to the baseline VHN_sound_ indentations. VHN_change_ values were calculated using the formula VHN_change_=100×(VHN_sound_−VHN_post_)/VHN_sound_.

### Transverse microradiography

One section, approximately 100 µm thick, was cut from the center of each specimen and across the specimen using a Silverstone-Taylor Hard Tissue Microtome (Scientific Fabrications Laboratories, USA). All sections were placed in the TMR-D1 v.5.0.0.1 system and X-rayed at 45 kV and 45 mA at a fixed distance of 12 s. An aluminum step wedge was X-rayed under identical conditions. Digital images were analyzed using the TMR software v.3.0.0.18. Sound enamel was assumed to be 87% v/v mineral. The data obtained from this analysis were integrated mineral loss (∆Z; %vol.μm) and lesion depth (L; μm).

## Statistical analysis

All three variables (VHN_change_, ∆Z, L) were analyzed using two-way ANOVA, with factors for sucrose concentration and surface conditioning as well as the interaction between them. All pair-wise comparisons from ANOVA analysis were made using Fisher’s Protected Least Significant Differences to control the overall significance level at 5%. Statistical analysis was performed using SAS version 9.4 (SAS Institute, Inc., Cary, NC).

## Results

The two-way interaction sucrose concentration × surface conditioning was not significant for VHN_change_ (p=0.872), ∆Z (p=0.662) or L (p=0.436). Surface conditioning affected VHN_change_ significantly (p=0.0079); however, it did not affect ∆Z (p=0.7383) or L (p=0.7323). Sucrose concentration impacted ∆Z (p<0.0001) and L (p<0.0001); however, it did not affect VHN_change_ (p=0.2877). Table 1 shows the data for all measured variables for each subgroup.

The VHN_change_ pairwise multiple comparison analyses indicated that the pellicle type created a significant difference between groups. In both sucrose concentrations, surface conditioning with filtered/pasteurized saliva resulted in the lowest VHN_change_ values, when compared with other surface conditioning groups. The difference between filtered/pasteurized subgroups and the two other surface conditionings was significant (filtered/pasteurized and filtered saliva subgroups p=0.006; filtered/pasteurized and DIW subgroups p=0.0075), while difference was insignificant between filtered saliva and DIW subgroups (p= 0.9312) (Table 1).

For the ∆Z values, the pairwise comparisons indicated a statistically significant difference only between 0.5% and 1% sucrose concentration (p<0.0001), and not based on the surface conditioning status. Growing the biofilm in 1% sucrose always resulted in lesions with higher ∆Z values, indicating more severe lesions.

Similarly, the pairwise comparisons for L values indicated a statistically significant difference between 0.5% and 1% sucrose (p<0.0001). Also, the L values were always higher in 1% incubation conditions, which means more severe carious lesions (Table 1).

## Discussion

This study aimed to evaluate the influence of surface conditioning using human saliva before biofilm formation *in vitro* on enamel demineralization. The statistical analysis results showed the hardness data were only affected by pellicle type, whereas the TMR data were only affected by sucrose concentration. To fully understand this contradiction, one should consider the differences between the variables studied.

Surface microhardness is a measurement of how a material responds to deformation. It is mainly influenced by surface integrity and rather than by structural characteristics or mineral content of the bulk substrate. One of the pellicle functions in the oral cavity is its masking effect: it coats dental surfaces and other structures, which may lead to different patterns of bacterial biofilm formation according to the presence/absence or the quality of the pellicle.^23-25^ The presence or absence of a pellicle layer, therefore, will affect surface characteristics, and this may explain the significant differences between pellicle subgroups in our study. On the other hand, TMR measures are based on mineral content rather than structure. Therefore, the expectation is to observe differences only when carious lesions with different mineral contents and/or distributions form during demineralization.^26^

Surface microhardness testing is straightforward and nondestructive. In some studies, it is coupled with transverse microradiography based on their objective. The minerals loss within the outer enamel was found to be proportional with the degree of the indenter penetration. However, deeper lesions cannot be quantitatively measured using surface microhardness.^27^ Moreover, surface microhardness is most effective in analyzing homogenous materials and shallow lesions only (e.g. enamel outer surface).^28^ White^28^ (1987) reported, in a study in which they evaluated the differences between surface microhardness and microradiography, that surface microhardness could detect remineralization in early lesions (or at least hardening of the surface without remineralization).^28^ Evaluating mineral content within the outermost layers of the enamel using microradiography is difficult. Therefore, the two analyses are usually considered complementary to each other in demineralization/remineralization studies.^28^

In this study, an active attachment model adopted from a previously published model was used.^1,20^ Despite still lacking more complex features that lead to more clinical relevance (e.g. pulsation of nutrients into the environment),^29^ an active attachment has the advantage of ensuring that the bacterial layers formed over the surface are not just sedimented cells, but rather attached to the enamel surface and to each other.^20^

A salivary bacterial mix was used to create a largely undefined microcosm biofilm. Before bacterial inoculation, the pooled saliva was tested for the presence of *Streptococcus mutans* and *Lactobacilli* using selective agars. The results confirmed the presence of both species. *In vitro* studies use various approaches with biofilms formed from monospecies (such as *Streptococcus mutans* or *Lactobacilli*),^20,30^ two or multiple species (3–10 species),^1,31^ or a microcosm biofilm.^32,33^ While single or multiple, defined species allow for greater control, employing a microcosm biofilm can result in greater clinical relevance. The acquired pellicle can be formed from saliva or plaque samples collected and pooled from single and/or multiple donors.^21^ This study used an approach based on conclusions drawn from previous studies.^21,32^ Some studies limited their salivary (or plaque) mix to be collected from a single donor,^33^ other studies collected samples from two or more donors.^34^ In this study, wax–stimulated saliva samples from three donors were pooled, thereby increasing the translational value of the findings.

One could suggest that using a microcosm biofilm source may result in large variability. However, the variability of biofilm characteristics in *in vitro* studies was explored previously,^21^ and most studies^33-35^ concluded that collection of saliva samples from the same donor at different times does not affect biofilm diversity. Moreover, the involvement of sucrose over time can lead to a dominance of certain bacterial strains (mainly cariogenic bacteria), thus overcoming initial differences between different samples (either from different donors or collected at different times from the same donor).^21^

The formation of acquired pellicle in *in vitro* studies can be conducted by exposing the surface of interest to sterile saliva solution for a certain period. Although including salivary pellicle in the model seems to be more clinically relevant, this step requires an expensive and time–consuming saliva sterilization (to ensure a bacterium–free solution that still contains salivary glycoproteins). Pasteurization was the method chosen to sterilize the diluted, pooled saliva samples.^22^ Although the samples were exposed to 60ºC for 30 minutes, this method still preserved salivary glycoproteins, as the heat needed for irreversible protein denaturation is at least 80ºC.

It was documented previously that the time required to form the pellicle *in vitro* ranges from 3 minutes to 7 days. The same studies reported minor relevance of pellicle maturation (i.e., aging).^36-38^ Based on that, the choice was to incubate the enamel samples in three surface conditioning media types for 5 minutes in this study. Exposing enamel surface *in vitro* to 1:10 diluted saliva for 5 minutes is still expected to maintain clinical relevance; the longer exposure to glycoproteins overcomes the dilution factor.

The second variable explored was sucrose concentration. Carbohydrate concentration within the growth media has been reported to impact the biofilm composition.^39^ Consequently, the biofilm cariogenicity may also be affected. As mentioned before, acquired pellicle formation is an integral step that precedes bacterial attachment to dental and oral surfaces. The formation of acquired pellicle generally consists of two stages.^40^ The first stage is very rapid and includes adsorption of salivary glycoproteins to the substrate. However, the second stage occurs immediately after the first stage *in vivo*.^40^ It is characterized by more adsorption of biomolecules, being the oral fluids the source of these biomolecules.^40^ Therefore, two different conditions of sources for the salivary pellicle (filtered/pasteurized and filtered saliva) were included in this study to represent these two stages and explore their influence in the pattern of demineralization. Although salivary pellicle formed from filtered/pasteurized saliva (which becomes free of viable bacteria) makes the *in vitro* study more controllable and the model more applicable if used in studies involving single/multiple species biofilm, using filtered saliva ensures more clinical relevance as the only eliminated element is food debris.

This study focused mainly on pellicle involvement in *in vitro* microbial studies, and on the influence of this factor on the hard tissue substrate characteristics. It did not test the influence of the presence of acquired pellicle on the cariogenicity of a microcosm biofilm. This can be tested in a similar study by collecting 48–hour biofilm and analyzing cariogenicity (e.g., lactic acid production). The bacterial source (i.e., saliva vs. plaque samples) may also be tested, since it was already reported that biofilms formed from saliva vs. plaque have different characteristics.^21^ Furthermore, different incubation times may affect pellicle formation and maturation. Lastly, pellicle formation can also be achieved by exposing specimens to the oral cavity for different periods of time, which provides material for future research. Lastly, all the variables tested may be evaluated in a prolonged study (more than 48 h) to observe the lesion characteristics, especially TMR data.

## Conclusion

Considering the limitations of this study, the presence or absence of an artificially induced acquired pellicle layer does not influence biofilm–mediated enamel caries lesion formation as measured by TMR. Some differences were observed using surface microhardness, indicating a complex interaction between pellicle proteins and biofilm–mediated demineralization of the enamel surface.

**Table 1:** Percentage surface microhardness (VHNchange), mineral loss (∆Z; %volmin.μm), and lesion depth (L; μm) data (mean ± standard deviation) for all treatment groups

Surface Conditioning																		
Sucrose Concen- tration	VHNchange						∆Z						L					
	Filtered/ Pasteurized Saliva		Filtered Saliva		Control (DIW)		Filtered/ Pasteurized Saliva		Filtered Saliva		Control (DIW)		Filtered/ Pasteurized Saliva		Filtered Saliva		Control (DIW)	
0.5%	32.7	a, A	51.5	b, A	48	b, A	820	a, A	1257	a, A	867	a, A	38.8	a, A	47.8	a, A	37.3	a, A
	±20.7		±18.4		±17.1		±266		±406		±338		±9		±9.6		±14.2	
1%	24.8	a, A	43.9	b, A	46.2	b, A	2623	a, B	2505	a, B	2428	a, B	78.4	a, B	73.3	a, B	74.6	a, B
	±22.4		±21		±18.8		±1014		±1480		±1208		±17.5		±26.7		±20.6	

Upper case letters indicate statistically significant differences between surface conditioning methods within sucrose concentrations

Lower case letters indicate statistically significant differences between sucrose concentrations within surface conditioning methods
